# Altered Global Signal Topography in Alcohol Use Disorders

**DOI:** 10.3389/fnagi.2022.803780

**Published:** 2022-02-16

**Authors:** Ranran Duan, Lijun Jing, Yanfei Li, Zhe Gong, Yaobing Yao, Weijian Wang, Yong Zhang, Jingliang Cheng, Ying Peng, Li Li, Yanjie Jia

**Affiliations:** ^1^Department of Neurology, The First Affiliated Hospital of Zhengzhou University, Zhengzhou, China; ^2^Department of Magnetic Resonance Imaging, The First Affiliated Hospital of Zhengzhou University, Zhengzhou, China; ^3^Engineering Technology Research Center for Detection and Application of Brain Function of Henan Province, The First Affiliated Hospital of Zhengzhou University, Zhengzhou, China; ^4^Key Laboratory of Magnetic Resonance and Brain Function of Henan Province, The First Affiliated Hospital of Zhengzhou University, Zhengzhou, China; ^5^Key Laboratory of Brain Function and Cognitive Magnetic Resonance Imaging of Zhengzhou, The First Affiliated Hospital of Zhengzhou University, Zhengzhou, China; ^6^Department of Neurology, Sun Yat-sen Memorial Hospital, Sun Yat-sen University, Guangzhou, China; ^7^Department of Anesthesiology, Beijing Friendship Hospital, Capital Medical University, Beijing, China

**Keywords:** alcohol-related cognitive impairment (ARCI), functional MRI, global signal topography, static GST, dynamic GST

## Abstract

The most common symptom of patients with alcohol use disorders (AUD) is cognitive impairment that negatively affects abstinence. Presently, there is a lack of indicators for early diagnosis of alcohol-related cognitive impairment (ARCI). We aimed to assess the cognitive deficits in AUD patients with the help of a specific imaging marker for ARCI. Data-driven dynamic and static global signal topography (GST) methods were applied to explore the cross-talks between local and global neuronal activities in the AUD brain. Twenty-six ARCI, 54 AUD without cognitive impairment (AUD-NCI), and gender/age-matched 40 healthy control (HC) subjects were recruited for this study. We found that there was no significant difference with respect to voxel-based morphometry (VBM) and static GST between AUD-NCI and ARCI groups. And in dynamic GST measurements, the AUD-NCI patients had the highest coefficient of variation (CV) at the right insula, followed by ARCI and the HC subjects. In precuneus, the order was reversed. There was no significant correlation between the dynamic GST and behavioral scores or alcohol consumption. These results suggested that dynamic GST might have potential implications in understanding AUD pathogenesis and disease management.

## Introduction

Uncontrolled and persistent alcohol consumption can exert a multitude of adverse health effects, such as chronic liver and kidney damages, and is considered a global public health concern. Alcohol use disorder (AUD) or alcoholism is a widespread health burden worldwide, affecting individuals at any level of socio-economic status. Studies have shown that AUD is considerably more prevalent in men than women in developing countries, resulting in an increased psychological and economic burden (Carvalho et al., 2019; Rehm and Shield, 2019). Like dose-dependent toxicity in most fatal health problems, heavy consumption of alcohol is directly related to an increased risk of developing AUD-linked multi-organ disorders (Schuckit, 2009). AUD is characterized by the emotional and/or psychological inability to resist oneself binge or heavy drinking habits, despite the known serious side-effects of excessive or persistent alcohol drinking (Wilcox et al., 2015). Interestingly, cognitive impairment has been frequently observed (~30–80%) in individuals undergoing AUD treatment (Bruijnen et al., 2019, 2021). It has been found that chronic alcohol abuse-induced thiamine deficiency serves as the major pathological contributor to AUDs, including alcohol-related brain damage (ARBD), with the symptomatic manifestation of cognitive deficits, apathy, severe memory deficits, vision impairment, and confabulations (Toledo Nunes et al., 2019; Kim et al., 2020; Oey et al., 2021). However, patients suffering from alcohol-related cognitive impairment (ARCI; Heirene et al., 2018) themselves do not lodge subjective complaints, possibly due to the lack of judgment ability under the influence of addiction (Walvoort et al., 2016). On the other hand, it has been shown in the clinical literature that cognitive deficits indulge addictive behaviors (Melugin et al., 2021). Moreover, cognitive impairment can also contribute to the lack of self-control in maintaining abstinence from longstanding alcohol abuse. Above all, many of the deficits associated with heavy alcohol consumption are potentially curable by abstinence (Ridley et al., 2013). However, the pattern and rate of cognitive recovery are not yet fully understood (Bates et al., 2002). Early identification of ARCI would be of major interest and potentially useful in daily clinical practice. There is an urgent need to explore the underlying pathophysiological processes and identify biomarkers.

Recently functional MRI (fMRI) studies have revealed that substance use (such as heroin, cigarette, and alcohol) can modulate spontaneous neuronal activity in the brain (Myrick et al., 2008; Franklin et al., 2011; Langleben et al., 2014; Cabrera et al., 2016). Furthermore, resting-state fMRI (rs-fMRI) has been evolved as an efficient diagnostic tool to examine AUD-induced neural pathogenesis. Aberrant brain functional connectivity patterns in the executive and salience mode network (ESMN) as well as default mode network (DMN) have been found across different categories of AUD and are associated with cognition and behavior-related disorders (Zhang and Volkow, 2019). Heavy alcohol drinking, a characteristic hallmark of AUD, can induce neurodegeneration, leading to long-term cognitive and behavioral dysfunctions that may transform one’s social drinking habit to addiction (Marshall et al., 2020). Alcohol addiction causes severe damage to the motivational circuit in three successive steps—exaggerated compulsive habit formation, overwhelming stress with lack of rewards, and compromised physical executive functions (Koob and Volkow, 2016). It has been delineated that chronic AUD can induce communication malfunction across large-scale brain networks (Zilverstand et al., 2018). Therefore, we exploited global signal topography (GST) to explore the cross-talks between local and global neuronal activities (Yang et al., 2017; Han et al., 2019; Zhang et al., 2019).

Although the rs-fMRI global signal data is contaminated by physiological noise and artifacts, however, recent studies have suggested that these data provide information about widespread neuronal activities (Power et al., 2014; Liu et al., 2017; Li et al., 2019). Furthermore, a direct relationship between GS and neural activity modulation has been established by the correlation of GS with local field potential readout at any certain position in the cerebral cortex (Schölvinck et al., 2010; Han et al., 2019). Hence, significant research studies were carried out to develop GST to analyze the correlation between local and global signals in the rs-fMRI study. It has been observed that the lower GST correlates with different cortical locations, such as the higher-order cortices and sensory cortex (Ao et al., 2021). Moreover, GST can be modulated by individual factors, like conscious state and attention-demanding tasks (Ao et al., 2021). In addition, abnormal GST has been detected in patients with bipolar disorder, schizophrenia, epilepsy, and major depressive disorder (Li et al., 2019, 2021; Wang et al., 2021). Together, these findings suggest a mechanistic insight into how GST and local neural activity coordinate to organize layers of information in the brain. Therefore, investigating the altered GST dynamics in AUD patients may provide potential insight into neural pathogenesis.

Here, we tested the hypothesis that the altered dynamic and static GST might play roles in the AUD neuro-pathogenesis and progression on three experimental groups. The dynamic GST measured the variations of dynamic coordination processes during scanning, while static GST indicated the coordination between global and local neuronal activities.

## Materials and Methods

### Participants

The Medical Ethics Committee of the Zhengzhou University’s First Affiliated Hospital approved the study protocol in compliance with the Helsinki Declaration, as revised in 2008. All participants were required to provide their signed written informed consent prior to completing the survey.

Individuals with AUD were recruited from a variety of sources, including the inpatient ward, internet posting, and advertisements. The control participants were volunteers from the local communities.

Inclusion criteria for recruitment of the participants were: (1) meeting the fifth edition of the Diagnostic and Statistical Manual of Mental Disorders (DSM-5) for moderate to severe AUD, as clinically assessed by the principal investigator; (2) on average, drinking more than 14 units of alcohol every week, according to the U.K. Chief Medical Officers (Health, 2017; Hawkins and McCambridge, 2021); (3) having Clinical Institute Withdrawal Assessment-Alcohol scale Revised (CIWA-Ar) score <9; and (4) able to understand and give written consent to study procedures.

Exclusion criteria for both healthy control (HC) and AUD patients were: (1) having a history of addiction, psychiatric disorder, and neurological and/or physical disorders that could influence brain morphology; (2) having contraindications in MRI; and (3) reportedly receiving interfering medications.

Cognitive dysfunctions were assessed on the Mini-Mental State Examination (MMSE) and Montreal Cognitive Assessment (MoCA) scales. Participants’ age ranged between 18 and 65 years. The Structured Clinical Interview (SCID)-non-patient edition was applied to screen the control subjects (Shabani et al., 2021) to confirm that they had no existing or previous mental disorders. On the other hand, AUD subjects were selected based on the Alcohol Dependence Scale (ADS) assessment, CIWA-Ar, Obsessive Compulsive Drinking Scale (OCDS), Visual Analog Scale (VAS), Pittsburgh Sleep Quality Index (PSQI), and Patient Health Questionnaire -9 (PHQ-9).

### Neuroimaging Data Acquisition

SIEMENS 3.0T scanner (MAGNETOM Prisma, Siemens, Germany) with a 16-channel head coil was used to obtain the fMRI data at the First Affiliated Hospital of Zhengzhou University. All participants were instructed to keep their eyes closed. Earplugs and foam padding were used to control head movements. At the end of scanning, subjects were reviewed whether they fell asleep anytime during the scanning. First, 3D anatomical T1-weighted magnetization-prepared rapid gradient echo (MPRAGE) MRI data with pulse sequence (TR 2,000 ms, TE 2.06 ms, T1 = 900 ms, the field of view 256*256 mm^2^, flip angle 9°, slice thickness 1.0 mm, matrix 256*256, 176 single-shot interleaved slices with no gap with isotropic voxel size 1*1*1 mm^3^) were obtained for all participants. Then, the rs-fMRI data were obtained using the parameters like TR 1,000 ms, TE 30 ms, the field of view 220*220 mm^2^, slice thickness 2.2 mm, slice gap 0.4 mm, flip angle 70°, and voxel size 2.0*2.0*2.2 mm^3^, with 52 slices and 400 dynamics. The slices aligned along the anterior and posterior commissure lines (AC-PC) were acquired with a total scan time of 360 s.

### Pre-processing

The CAT12 toolbox^1^ was used to pre-process all T1-weighted images using standard pipeline steps. After checking the image artifacts, the image origin was adjusted to the AC line. Image normalization was carried out as per the Montreal Neurologic Institute’s space and segmentation models into white matter (WM), gray matter (GM), and cerebrospinal regions. Then, images were reassigned to a volume-image resolution of 1.5 × 1.5 × 1.5 mm^3^. At the final step, the GM maps were relapsed using 6 mm full width at half maximum (FWHM) Gaussian kernel.

The Data Processing Assistant for Resting-State fMRI analysis toolkit (DPARSF) was used to pre-process rs-fMRI data. The first ten volumes were discarded, and the slice-timing was adjusted accordingly, along with realignment. Then image normalization was performed with respect to the standard EPI template (resampled into 3*3*3 mm^3^). Participants were excluded if the head motion was >3 mm in maximum displacement or >3° rotation in angular motion. No participant was excluded in this step. Then images were smoothed using a 6*6*6 mm^3^ FWHM Gaussian kernel and readjusted to minimize the low-frequency drift. Finally, the band-pass filter (0.01–0.1Hz) removed the high-frequency physiological noises. Friston 24-motion parameter model was applied to obtain signals from the cerebrospinal fluid (CSF) and WM areas, which were relapsed as noise covariates. The outliners were despiked using the 3rd order spline fit to clean the respective segments of the time course. Outliners were identified using AFNI 3dDespike^2^.

### Voxel-Based Morphometry (VBM)

Spatial segmentation and normalization were applied into three voxel classes: GM, WM, and CSF, using volume segmentation and adaptive maximum a posterior (MAP) filtering approach. Regional GM volume differences were tested using normalized GM maps. Then the obtained GM maps were flattened using a 6 mm FWHM Gaussian kernel for further analysis. The 0.1 absolute masking threshold was applied to the VBM data.

### Statistical Analysis

#### Analysis of the Static GST

The static GST was measured by pairwise time course correlation between the GS and each voxel segment. The GS time series was calculated as the mean pre-processed BOLD signal, averaged over all GM voxels for each of the time points, excluding WM and ventricle signals. Fisher’s r-to-z transformation was applied to convert correlation coefficients into *z* scores. Here, the correlation map was constructed by the FC.

#### Analysis of the Dynamic GST

The dynamic GST correlation was calculated using a sliding window-based method, as described elsewhere (Allen et al., 2014). The length of the rectangular window was set to 22 TRs (44 s) with a step size of 1 TR. Pearson correlation coefficient was first computed for each sliding window between the time series and GST for each voxel in the GM, and then the coefficient was transformed into Fisher’s *z*-score. The standard deviation (SD) of the dynamic GST correlation of each voxel was then calculated. To eliminate the influences of parameter selection, the window length of 30 TRs with a step size of 1 TR was applied.

## Results

### Participants

The study cohort consisted of 40 HCs, and 80 patients with AUD. The recruited AUD patients were divided based on their MMSE and MoCA scores into the cognitive impairment group (ARCI; MMSE<24 and MoCA<26) and non-cognitive impairment group (AUD-NCI; MMSE≥24 and MoCA≥26). Among the AUD subjects, 26 patients exhibited cognitive impairment. The years of alcohol consumption of the AUD group participants ranged from 17 to 23 years. The mean drinking frequency was 4.8 times per week. The median total quantity of pure alcohol consumption was 325.262 kg. The ADS score of the AUD group was 26.06 ± 3.55 (*p* < 0.05).

The MoCA and MMSE scores were lower in the AUD group than in the HC group (*p* > 0.05), and the ADS, CIWA-Ar, and OCDs scores were higher in the AUD group than in the HC group (*p* < 0.05). The high voiceover signal in the lateral ventricle score and encephalatrophy (GCA) in the AUD group were higher than in the HC group (*p* > 0.05). The detailed demographic information and clinical characteristics are presented in Table 1.

**Table 1:** Patient demographics and clinical characteristics.

	Control group (*n* = 40)	AUD group (*n* = 80)	*p*
**General character**
Age (y)	48.63 ± 7.56	48.12 ± 6.64	0.084
Sex (F%)	1 (2.5%)	1 (1.25%)	0.396
Years of schooling (y)	9.4 ± 3.3	9.01 ± 2.9	0.693
Height (cm)	174.50 ± 5.26	172.91 ± 3.47	0.350
Weight (Kg)	70.50 ± 4.38	76.05 ± 3.47	0.095
Time of alcohol drinking	0.24 ± 0.74	20.51 ± 2.78	0.000*
Pure alcohol (g, total)	629.99 ± 192.17	325,262.37 ± 30,806.74	0.001*
**Characteristics of performance in multidimensional neuropsychological battery**
MMSE	29.6	27.4	0.061
MoCA	26.3	24.4	0.058
ADS	1.50 ± 0.74	26.06 ± 3.55	0.001*
CIWA-Ar	0.51 ± 0.12	8.15 ± 2.74	0.009*
OCDs	2.23 ± 1.67	12.67 ± 2.57	0.000*
VAS	1.63 ± 0.74	2.12 ± 0.89	0.053
PHQ-9	5.32 ± 0.1.74	6.79 ± 1.52	0.065
**Characteristics of various MRI-defined lesions in CSVD participants**
High voiceover signal in the lateral ventricle (score)	1.7	1.9	0.071
Encephalatrophy (GCA)	1.2	1.4	0.34

*p < 0.05.

**Figure 1:**
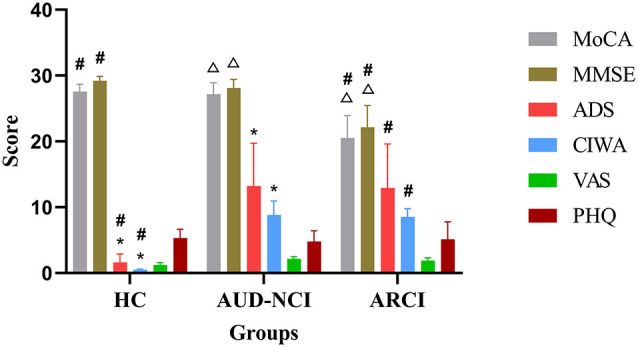
Cognitive assessment scales compared in these three groups. *p < 0.05 HC group vs. AUD-NCI group. ^#^*p* < 0.05 HC group vs. ARCI group. ^△^*p* < 0.05 AUD-NCI group vs. ARCI group. AUD, alcohol use disorder; NCI, non-cognitive impairment; HCs, healthy controls; ARCI, alcohol-related cognitive impairment.

We could not detect any significant differences across the groups with respect to age, sex, height, weight, education, and handedness in the demographic data. However, the AUD patients presented significantly higher ADS scores as well as the items of DSM-5 AUD Criteria (*p* < 0.05, Figure 1). The analysis of variance (ANOVA) method was used for the statistical analysis of the results.

### VBM Analysis of the AUD-NCI and ARCI Groups

There were no significant differences in total GM, WM, and CSF values between AUD-NCI and ARCI groups. VBM analysis between AUD-NCI and ARCI patients showed no significant differences using a whole-brain threshold of *p* < 0.05.

### Static GST in the HCs, and Patients With AUD-NCI and ARCI

There were no differences in the static GST values between HC, AUD-NCI, and ARCI groups as measured by ANOVA.

### Dynamic GST in AUD Patients

**Figure 2:**
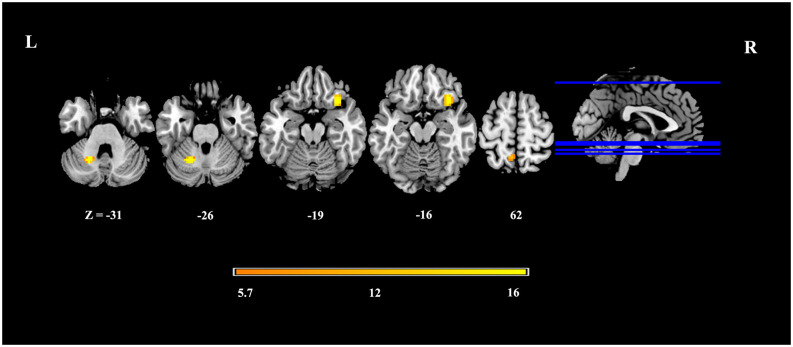
Altered dynamic GS topography in AUD patients.

**Figure 3:**
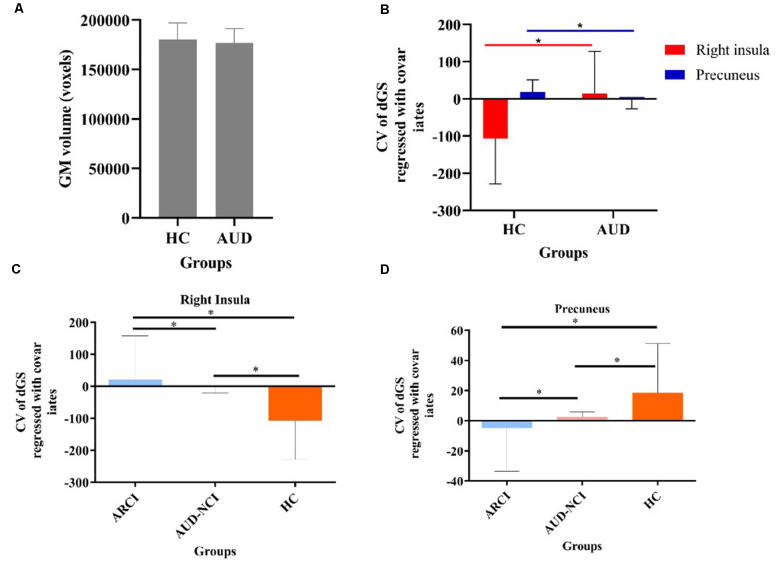
The static and temporal dynamic changes of intrinsic brain activity in control and AUD groups. **(A)** Gray matter (GM) volume in voxels between HC and AUD group (*p* > 0.05). **(B)** Dynamic global signal topography (GST; right insula and precuneus) in HC and AUD groups (*p* < 0.05). **(C)** Dynamic GST in the right insula in different groups (*p* < 0.05). **(D)** Dynamic GST in precuneus in different groups.*p < 0.05.

In dynamic GST measurements, control subjects had the lowest coefficient of variation (CV: SD/mean) at the right insula, followed by ARCI, and then the AUD-NCI patients. Notably, the CV order was found reversed in the precuneus region (Figures 2, 3, Table 2).

**Table 2:** Altered dynamic GS topography in AUD patients compared with HCs.

Clusters	Number of voxels	Regions	MNI	F
1	39	Cerebellum Anterior Lobe	−21, −57, −30	25.92
2	48	Right insula Inferior frontal gyrus	33, 18, −18	25.68
3	15	Parietal lobe Left Precuneus Postcentral gyrus	−6, −54, 66	22.60

*GS, global signal; AUD, alcohol use disorder; HCs, healthy controls*.

### Correlation Analyses

The correlation analyses showed no significant correlations between the dynamic GST and total quantity of pure alcohol, MMSE, MOCA, ADS, OCDS, VAS, or PHQ-9.

## Discussion

AUD is one of the most prevalent and devastating neurological and addictive disorders (Leclercq et al., 2020). Alcohol addiction can potentially increase the risks of suicidal tendency due to impaired judgment capacity, increased impulsivity related to mood disorders, psychotic disorders, anxiety, and cognitive deficits (Kudva et al., 2021). Cognitive dysfunction in AUD patients can affect their psycho-social behavior and therapeutic outcomes. However, the cognition assessment scales and the commonly used MRI sequences are not efficient enough to detect early-stage alcohol-induced damage. Hence, we aimed to assess the cognitive deficits in AUD patients through the development of a specific imaging marker for AUD-linked cognitive deficits.

Here, we aimed to explore the correlations between altered dynamic and static GST and cognitive deficits in AUD patients. This study investigated the modified large-scale brain functional organization in AUD using a data-driven image analysis method. Using a threshold of FWE-corrected *p* < 0.05, VBM analyses could not show any differences between the HC, AUD-NCI, and ARCI groups. There were also no significant differences in the static GST values between the HC, AUD-NCI, and ARCI groups as measured by ANOVA. Interestingly, for the dynamic GST measurements, we observed that HC group subjects had the lowest CV values at the right insula, followed by ARCI, and AUD-NCI subjects in the ascending order. Whereas in the case of the precuneus region, this order was reversed. According to the previous studies, there is a significant correlation between global signal amplitude and functional connectivity with the level of consciousness, irrespective of physiologic, pathologic, and pharmacologic etiologies. These two regions are known to be involved in the memory uptake and storage processes (Tanabe et al., 2020), which were changed in the dynamic GST analysis earlier than the cognitive scale assessment and fMRI examination. Collectively, these findings suggest that the GST is not static, rather dynamic, thus providing useful information about the pathophysiological mechanisms of AUD.

In line with previous findings, AUD patients exhibited impairments in the insula and precuneus regions (Fukushima et al., 2020; Bordier et al., 2021). Recent fMRI studies have demonstrated that AUD can enhance activation in the insula, inferior frontal gyrus, and precuneus (Noori et al., 2016; Quaglieri et al., 2020). The precuneus has been functionally linked to resting-state cognitive activities, including the evaluation and collection of information, extraction of episodic memory, emotion, and anxiety, and self-referred mental activity (Krienke et al., 2014). Furthermore, our study indicated that in AUD patients, functional alterations in the precuneus might affect the episodic memory-related circuits. A study has shown that insula as part of the salience network mediates external stimuli-induced attention and arousal responses (Bordier et al., 2021). The insula, as the central hub of the salience network, regulates attention shifting between the exogenous and endogenous states.

VBM studies have revealed AUD-induced atrophic patterns in the anterior and posterior cingulate cortices, lateral prefrontal cortex, hippocampus, insular-opercular cortex, striatum, and thalamus (Xiao et al., 2015; Yang et al., 2016; Galandra et al., 2018). However, in our cohort, we could not find any significant morphometric differences between the groups in the corresponding brain regions. However, there were significant differences in dynamic GST values across the groups, suggesting that dynamic GST could detect AUD-mediated changes before the manifestation of cortical atrophy.

According to the data, we found that the two indexes were different among the three groups. However, there was no obvious linear correlation between the amount of consumed alcohol and behavioral scores in AUD patients. The possible reasons were speculated as follows: (1) the sample size we included was not large enough. More participants are needed to find the correlation; (2) all the included patients were able to cooperate with the examination, but the cognitive function decline was not severe enough; (3) there are individual differences with respect to brain damage caused by regular alcohol consumption due to altered metabolic enzyme activity and other unknown mechanisms; and (4) the dynamic GST abnormality might have happened prior to behavioral scale changes in alcohol dependence.

There were several limitations in this study. First and most importantly, this study involved a very small sample size which limited our ability to detect effects on the statistical scale. In the future, we will plan to carry out studies involving a larger sample size with higher statistical significance, primarily focusing on the dynamic GST. Secondly, as the cohort consisted of only severe-to-moderate AUD patients, sampling bias should be taken into account. To confirm whether the BOLD activity was a state-dependent endophenotype, mild AUD patients should be examined using the same method. Thirdly, ADS score and alcohol consumption rate was evaluated based on the self-administered questionnaires that might increase the risk of possible recall bias and false positives. However, despite these limitations, this study is likely to broaden our understanding of alcohol addiction and its impact on neural network modulation to facilitate pathology detection and treatment strategy.

## Data Availability Statement

The raw data supporting the conclusions of this article will be made available by the authors, without undue reservation.

## Ethics Statement

The studies involving human participants were reviewed and approved by the Ethics Committee of First Affiliated Hospital of Zhengzhou University (2018-KY-91) and abided by the Declaration of Helsinki. The patients/participants provided their written informed consent to participate in this study. Written informed consent was obtained from the individual(s) for the publication of any potentially identifiable images or data included in this article.

## Author Contributions

YJ was the project holder. YJ and LL contributed to conception and study design. RD and LJ were responsible for study follow-up and contributed to this article. YL, ZG, and YY were responsible for patients’ recruitment, diagnosis, and treatment. WW was responsible for fcMRI acquisition. YZ, JC, and YP analyzed the data. All authors contributed to the article and approved the submitted version.

## Conflict of Interest

The authors declare that the research was conducted in the absence of any commercial or financial relationships that could be construed as a potential conflict of interest.

## Publisher’s Note

All claims expressed in this article are solely those of the authors and do not necessarily represent those of their affiliated organizations, or those of the publisher, the editors and the reviewers. Any product that may be evaluated in this article, or claim that may be made by its manufacturer, is not guaranteed or endorsed by the publisher.

## Footnotes

^1^ http://dbm.neuro.uni-jena.de/cat12/

^2^ https://afni.nimh.nih.gov/

## Abbreviations

AUD: alcohol-use disorders
ARBD: alcohol-related brain damage
ARCI: alcohol-related cognitive impairment
GST: global signal topography
HC: healthy control
VBM: voxel-based morphometry
MRI: magnetic resonance imaging
fMRI: functional magnetic resonance imaging
ESMN: executive and salience mode network
DMN: default mode network
DSM-5: diagnostic and statistical manual of mental disorders
CIWA-Ar: clinical institute withdrawal assessment-alcohol scale revised
MMSE: mini-mental state examination
ADS: alcohol dependence scale
OCDS: obsessive compulsive drinking scale
VAS: visual analog scale
PSQI: Pittsburgh Sleep Quality Index
PHQ-9: patient health questionnaire-9
MPRAGE: magnetization-prepared rapid gradient echo
WM: white matter
GM: gray matter
FWHM: full width at half maximum
DPARSF: the data processing assistant for resting-state fMRI analysis toolkit
CSF: cerebrospinal fluid
SD: standard deviation.
